# Evaluating ChatGPT-4o and OpenEvidence for Structural Heart Disease Support: Insights From Tricuspid Valve Therapies

**DOI:** 10.1016/j.shj.2025.100696

**Published:** 2025-07-05

**Authors:** Joseph Hajj, Mohamad Mdaihly, Joseph Kassab, Rhonda Miyasaka, Serge C. Harb

**Affiliations:** a Department of Cardiovascular Medicine, Heart and Vascular Institute, Cleveland Clinic, Cleveland, Ohio; b Department of Internal Medicine, UT Southwestern Medical Center, Dallas, Texas

**Keywords:** Artificial intelligence, ChatGPT-4o, OpenEvidence, Structural heart disease, Tricuspid valve interventions

## Abstract

• Evaluated ChatGPT-4o and OpenEvidence for structural heart disease decision support.
• Among the first to assess the use of artificial intelligence amid rising adoption of TriClip and EVOQUE for transcatheter tricuspid valve interventions.
• ChatGPT-4o delivered more accurate responses and proved more clinically reliable.
• Neither platform replaces clinical judgment; ChatGPT-4o excels in fast-paced settings.

Artificial intelligence (AI) tools such as ChatGPT and OpenEvidence are increasingly being explored as real-time clinical support systems. While prior research has assessed AI chatbot accuracy in areas such as preventive cardiology and hypertension management, these studies primarily evaluated earlier, outdated versions of ChatGPT that were not specifically designed for medical use and are undergoing rapid ongoing development.^1^^,^^2^ As a result, data remain limited, particularly in highly specialized fields such as structural heart disease.

This study is among the first to evaluate AI performance on complex structural heart topics, where rapidly evolving evidence and limited clinician familiarity underscore the need for accurate and up-to-date guidance. This is particularly timely given the growing clinical focus on tricuspid valve therapies, following the Food and Drug Administration’s 2024 approval of 2 transcatheter systems: the TriClip device (Abbott, Abbott Park, Illinois) for edge-to-edge repair in patients with severe symptomatic tricuspid regurgitation at high surgical risk and the EVOQUE system (Edwards Lifesciences, Irvine, California) for valve replacement in those at similarly elevated risk.^3^^,^^4^

As these therapies become more widely adopted, internal medicine residents, cardiology fellows, and general cardiologists are increasingly likely to seek quick, reliable answers to clinical questions and will be responsible for following up with patients treated with these novel interventions. Our study aimed to evaluate and compare the clinical accuracy of responses generated by ChatGPT-4o and OpenEvidence, with the latter gaining traction through its partnership with the New England Journal of Medicine and restricted to use by licensed physicians.

To reflect real-world information needs, we developed 15 clinician-facing questions focused on transcatheter tricuspid valve repair and replacement. These questions were submitted to both AI platforms in May 2025. Responses were then independently reviewed by 2 board-certified cardiac imaging specialists. Responses were rated as accurate when they were deemed complete and clinically appropriate, inaccurate if they were entirely incorrect, and partially accurate if they included some correct information but lacked completeness, based on the clinical judgment of the reviewing cardiologists (Table 1).

**Table 1:** Performance comparison of ChatGPT-4o and OpenEvidence in answering clinical questions on transcatheter tricuspid valve interventions

Question	OpenEvidence accuracy∗	Time for OpenEvidence answer (seconds)†	ChatGPT-4o accuracy∗	Time for ChatGPT-4o answer (seconds)†
1. My patient has severe TR and a pacemaker. Are they a candidate for transcatheter intervention?	Accurate	14	Accurate	2.3
2. My patient has severe TR and chronic kidney disease (CKD). Can they undergo transcatheter tricuspid valve intervention safely?	Accurate	14	Accurate	5.1
3. My patient has TR due to a flail leaflet following cardiac biopsy. Which transcatheter option is more appropriate, TEER or valve replacement?	Inaccurate	10.2	Inaccurate	2.2
4. My patient has severe TR but is asymptomatic. Should I refer them for transcatheter therapies?	Accurate with missing information	15.1	Accurate with missing information	2.1
5. My patient has severe TR and right ventricular failure. Is TEER preferred over replacement in this setting?	Inacurrate	14.3	Accurate	1.8
6. My patient has severe residual TR after undergoing TEER with TriClip. Are they a candidate for additional clipping? Are they a candidate for TTVR, and which is preferred?	Inacurrate	11.3	Accurate with missing information	1.8
7. My patient recently underwent successful transcatheter therapy for TR. Should I stop their diuretic therapy?	Accurate	20.6	Accurate	1.5
8. I am referring my patient for TEER, but their kidney function is compromised. Do they need a contrast-enhanced CT scan?	Accurate with missing information	16	Accurate	1.7
9. I am referring my patient for TTVR, but they have impaired kidney function. Is contrast-enhanced CT still necessary?	Accurate with missing information	13.5	Accurate	1.9
10. My patient is presenting after TTVR with a mean tricuspid valve gradient of 10 mmHg. What should I suspect?	Inaccurate	10.7	Accurate	1.8
11. My patient has a post-TTVR mean tricuspid gradient of 10 mmHg. What are the differentials and what workup should I order?	Inaccurate	10.5	Accurate with missing information	2.05
12. I am seeing a patient after TEER. Should they be on anticoagulation therapy?	Inaccurate	18.9	Accurate	2.3
13. I am seeing a patient after tricuspid valve replacement. Should they be on anticoagulation therapy?	Accurate	16.5	Accurate	1.6
14. My patient has a previously implanted tricuspid annuloplasty ring. Can they still undergo transcatheter repair or replacement?	Inaccurate	14.5	Accurate	1.5
15. My patient is elderly with severe TR. How does age influence the decision between repair and replacement?	Inaccurate	12.2	Inaccurate	1.96

Abbreviations: AI, artificial intelligence; CT, computed tomography; TEER, transcatheter edge to edge repair; TR, tricuspid regurgitation; TTVR, transcatheter tricuspid valve replacement.

∗ Each question was submitted in a separate chat session to avoid bias from prior interactions. Two experienced cardiologists independently rated the accuracy of each answer. In cases of disagreement, they discussed and reached a consensus rating together.

† Response time was measured using a smartphone stopwatch, started when the “Enter” key was pressed and stopped when the AI began generating a response.

Reviewer agreement was assessed using the free-marginal Fleiss kappa statistic (κ). When discrepancies in grading occurred, the cardiologists engaged in discussion and were given the chance to revise their assessments until they reached a mutual consensus.

We involved 2 expert reviewers to better reflect real-world clinical practice, where differences in judgment are common but typically reconciled through discussion and consensus. Throughout the evaluation process, the cardiologists were blinded to the source of each response, ensuring that their assessments were unbiased and based solely on the content’s quality.

Interrater agreement was high for both AI platforms. For ChatGPT-4o responses, the 2 cardiologists demonstrated κ = 0.87 (95% CI: 0.63-1.00), reflecting almost perfect agreement and an overall concordance rate of 93.3%. For OpenEvidence-generated answers, interrater reliability was also strong, with κ = 0.69 (95% CI, 0.39-0.99), indicating substantial agreement and an overall agreement of 86.7%.

In addition to accuracy, we evaluated the average time each platform required to begin generating responses, which revealed a statistically significant difference. ChatGPT-4o responded considerably faster, with a mean initiation time of 2.11 ± 0.84 seconds, compared to 14.15 ± 2.91 seconds for OpenEvidence (*p* < 0.001, Welch’s *t*-test). This notable difference highlights ChatGPT’s potential advantage in delivering timely information in clinical environments.

Following consensus between the expert reviewers, ChatGPT-4o provided 10 fully accurate responses (66.7%), 3 responses (20.0%) that were accurate with missing information, and 2 responses (13.3%) classified as inaccurate. In contrast, OpenEvidence produced only 4 fully accurate answers (26.7%), with 3 responses (20.0%) rated as accurate with missing information and 8 responses (53.3%) deemed inaccurate.

The likelihood of delivering a fully accurate response was significantly higher with ChatGPT-4o compared to OpenEvidence (relative risk [RR], 2.5; 95% CI, 1.02-6.14; *p* = 0.043), underscoring its potential value as a more reliable clinical decision support tool in the context of tricuspid valve interventions.

Our findings reveal meaningful differences in the performance and clinical utility of 2 prominent AI platforms, ChatGPT-4o and OpenEvidence, when addressing complex questions related to tricuspid valve interventions. ChatGPT-4o demonstrated higher accuracy, significantly faster response times, and greater interreviewer consistency, suggesting it may serve as a more reliable and efficient tool for point-of-care education and clinical decision support. This distinction is especially important in structural heart disease, where novel therapies such as TriClip and EVOQUE have only recently received Food and Drug Administration approval and where trainees and general cardiologists are likely to seek accessible, up-to-date information.

At the same time, our results underscore that neither platform can yet be fully trusted without expert oversight. Both tools showed limitations in completeness and accuracy, reinforcing the need for their use as adjuncts rather than replacements for clinical judgment. When rapid answers are needed in busy clinical environments, ChatGPT-4o appears more suitable due to its responsiveness and adaptability.

Although not explicitly tested in this study, our experience highlighted important functional differences. A notable strength of OpenEvidence is its integration of citation-linked, up-to-date references, an asset for clinicians involved in academic writing, literature reviews, or manuscript preparation. This feature makes OpenEvidence particularly well-suited for research-oriented tasks. However, its reliance on well-structured and precise queries may limit its practical utility in real-time clinical scenarios. In contrast, ChatGPT-4o exhibited strong contextual awareness, consistently interpreting vague, incomplete, or grammatically flawed questions with ease, an advantage that enhances its usability in fast-paced, real-world settings where flexibility and immediacy are essential.

This study has several important limitations. First, although accuracy was assessed through expert consensus, the grading process was inherently subjective and not based on a standardized or validated rubric. Second, as both AI platforms are continuously evolving, the findings represent a snapshot of their performance at a single time point (May 2025), which may limit reproducibility over time. Lastly, as with any emerging clinical technology, a thorough evaluation of safety, efficacy, and downstream impact is essential before recommending widespread adoption in medical decision-making.

## Declaration of Generative AI and AI-Assisted Technologies in the Writing Process

During the preparation of this work, the authors used ChatGPT-4o and OpenEvidence as sources of data by querying each platform with a standardized set of clinician-facing questions related to transcatheter tricuspid valve interventions. The AI-generated responses were collected in May 2025 and analyzed for clinical accuracy, response time, and usability. The authors did not use AI tools to assist with writing or editing the manuscript and take full responsibility for the content and interpretation of the findings.

## Ethics Statement

This study was conducted in accordance with institutional ethical standards and relevant national and international guidelines. As the research involved publicly available large language models and did not include human subjects, identifiable patient data, or intervention, formal IRB approval was not required.

## Funding

The authors have no funding to report.

## Disclosure Statement

S. C. Harb serves as a consultant, speaker, or advisory board member for the following companies: TeraRecon, Inc; Vahaticor, Inc; Abbott Laboratories; Boston Scientific Corporation; and Edwards Lifesciences LLC. R. Miyasaka is a consultant and/or speaker for Abbott Laboratories. All relevant disclosures have been reported; however, none are related to or have influenced the content of this manuscript. The other authors had no conflicts to declare.
